# Effects of* Lycium barbarum* Polysaccharides on Apoptosis, Cellular Adhesion, and Oxidative Damage in Bone Marrow Mononuclear Cells of Mice Exposed to Ionizing Radiation Injury

**DOI:** 10.1155/2016/4147879

**Published:** 2016-05-26

**Authors:** Jing Zhou, Hua Pang, Wenbo Li, Qiong Liu, Lu Xu, Qian Liu, Ying Liu

**Affiliations:** Department of Nuclear Medicine, The First Affiliated Hospital of Chongqing Medical University, Chongqing 400016, China

## Abstract

*Lycium barbarum* has been used for more than 2500 years as a traditional herb and food in China. We investigated the effects of* Lycium barbarum* polysaccharides (LBP) on apoptosis, oxidative damage, and expression of adhesion molecules in bone marrow mononuclear cells (BMNC) of mice injured by ionizing radiation. Kunming mice were exposed to X-rays; then mice in the LBP groups were continuously injected with various concentrations of LBP intraperitoneally for 14 days. Mice in the control group were continuously injected with normal saline (NS) by the same route for 14 days. A normal group was set up. After 1, 7, and 14 days of treatment, mice were killed and BMNC were extracted. Cell cycle, apoptosis, and the expression of adhesion molecules CD44 and CD49d were detected by flow cytometry. The levels of malondialdehyde (MDA) and superoxide dismutase (SOD) were identified by colorimetric analyses. LBP significantly decreased the percentage of G_0_/G_1_ phase, apoptosis, MDA level, and expression of CD44 and CD49d and distinctly increased the activity of SOD. LBP showed a protective effect on BMNC against ionizing radiation-induced apoptosis and oxidative damage and altered the expression of adhesion molecule.

## 1. Introduction

The impact of ionizing radiation on humans is an increasingly serious issue. The degree of damage to human health is closely related to the dose of radiation and duration of exposure. In addition to a direct effect on the biological macromolecules, causing damage to DNA molecules via single or double chain rupture and depolymerization, the radiation decreases or inactivates enzymatic activity. The cell membrane is a direct target and cell biology is also altered as a whole indirectly [1]. It adversely affects every organ and system, especially hematopoietic system and nervous system. Ionizing irradiation also confers benefits for humans in equal measure. With advances in nuclear technology, exposure to radiation such as X-rays, CT, and other cancer therapies has become inevitable [2]. Therefore, the protection of normal tissue during actinoscopy or radiotherapy is an important therapeutic goal and the focus of radioprotection has become increasingly therapeutic [3]. Currently, there are several antiradiation drugs such as sulfhydryl-ammonia compounds, cytokines, and hormones, which are clinically unsatisfactory due to restricted treatment range, toxic side effects, and high price [4]. Consequently, a search for nontoxic or low-toxic antiradiation drugs derived from traditional herbs has become imperative.* Lycium barbarum* (Gouqi) belongs to the plant family Solanaceae. The red berries have been used as a traditional herb and food supplement for more than two thousand years, including medicinal beverages and healthy soups.* Lycium barbarum* contain abundant polysaccharides, scopoletin, the glucosylated precursor, and stable vitamin C analog 2-O-*β*-d-glucopyranosyl-l-ascorbic acid, carotenoids, betaine, cerebroside, *β*-sitosterol, flavonoids, amino acids, minerals, and vitamins.* Lycium barbarum* polysaccharide is the main component of these biological activities, with a relative molecular mass of 68–200 kDa [5]. It has multiple biological and pharmacological functions, such as anticancer [6–12], antifatigue [13], neuroprotective [14, 15], antioxidant [16, 17], hypoglycemic [18–20], fertility-protective [2, 21], and immunomodulating [22]. Recent studies show that LBP has a protective effect against ionizing radiation-induced damage; LBP can protect the reproductive function and prevent spermatogenic cell apoptosis induced by irradiation and enhance self-repair of the testis [2, 3, 23, 24]. It also can promote the immune function recovery of radiated mice [25]. Hematopoietic system is also a radiative sensitive target organ and leukopenia is the most classic radiation damage index; there is a sharp drop in white blood cells after irradiation; the indexes of the thymus and spleen of mice also significantly reduced; myelosuppression can be induced, but LBP can effectively improve the situation [5, 26, 27]. In our study, Kunming mice were exposed to X-rays to establish radiation injury models. After 1, 7, and 14 days of treatment, the percentage of G_0_/G_1_ phase, apoptotic rate, the expression of CD44 and CD49d, MDA content, and SOD activity in BMNC were measured. LBP played an effective role in protecting bone marrow mononuclear cells against ionizing radiation injury.

## 2. Materials and Methods

### 2.1. Preparation of* Lycium barbarum* Polysaccharides

Purified Lycium barbarum polysaccharides (LBP) were purchased from Shanghai Kang Zhou Funqi Polysaccharide Co., Ltd. (Shanghai, China), which were freeze-dried into powder for storage. The freeze-dried powder of LBP was immediately diluted with double distilled water for experimental use.

### 2.2. Reagents

Lymphocyte separation medium kit was obtained from TBD Co., Ltd. (Guangdong, China). Annexin V-FITC/PI detection kit and BCA Protein Assay Kit were obtained from Beyotime Biological Technology Research Institute (Jiangsu, China). P-Phycoerythrin- (PE-) anti-mouse Sca-1^+^ antibodies and isotype control antibody were obtained from eBioscience Corporation (Shanghai, China).

Fluorescein isothiocyanate- (FITC-) anti-mouse CD44 antibodies and anti-mouse CD49d antibodies as well as isotype control antibody were obtained from eBioscience Corporation (Shanghai, China). Fluorescein isothiocyanate- (FITC-) anti-mouse CD49d antibodies and isotype control antibody were obtained from eBioscience Corporation (Shanghai, China). Superoxide dismutase and maleic dialdehyde test kits were obtained from Nanjing Jiancheng Bioengineering Institute (Nanjing, China).

### 2.3. Experimental Grouping and Treatment

Thirty-nine male and thirty-nine female Kunming mice weighing 18–22 g were included in this study. Mice were provided by the Animal Experiment Center of Chongqing Medical University. Mice were housed and fed for 14 days under a 12 h light/dark photoperiod at an appropriate temperature and humidity. The mice were randomly divided into 5 groups: normal control, NS control, 50 mg/kg LBP, 100 mg/kg LBP, and 200 mg/kg LBP [28]. Except normal control group, mice in the other groups were exposed to 4.0 Gy X-rays for 1.25 minutes once using a linear accelerator (2300 CD, Oncology Department, the First Affiliated Hospital of Chongqing Medical University, Chongqing, China). X-ray dose absorption rate was about 3.76 Gy/min. At an irradiation target distance of 100 cm, the irradiated area was about 25 cm × 25 cm [29]. Within 2 h after irradiation, mice in the experimental groups were continuously injected with 50 mg/kg LBP, 100 mg/kg LBP, and 200 mg/kg LBP intraperitoneally for 14 days, respectively. Mice in the control group were continuously given NS similarly for 14 days. Before each injection, the weight of mice was measured, and the injection doses were based on actual body weight. After 1, 7, and 14 days of treatment, mice were killed and BMNC were extracted [2, 3], and various indices were examined.

### 2.4. Preparation of BMNC

After 1, 7, and 14 days of treatment, mice were killed by cervical dislocation to severe bilateral femur. After removal of the connective tissues and muscles, BMNC were flushed rapidly out of the femur using PBS buffer solution and filtered by size four needle as a monoplast suspension in a 10 mL centrifuge tube. After centrifugation at 950 rpm for 10 min, the supernatant was discarded. A moderate amount of PBS buffer solution was added to scattered cells to obtain bone marrow cell suspension. BMNC were acquired using Ficoll-Hypaque density gradient centrifugation as follows. First, 2 mL bone marrow cell suspension was dropped slowly along the wall of the centrifuge tube on 5 mL lymphocyte separation medium with a transfer pipette. Second, after a 25 min centrifugation at 950 rpm, the white film of BMNC was carefully aspirated along the wall of centrifuge tube into another clean centrifugal tube. Finally, BMNC were washed twice with PBS buffer solution.

### 2.5. Determination of Cell Cycle

After 1 to 14 days of treatment, the BMNC suspension was isolated and fixed in ice-cold 70% ethanol overnight at 4°C. Next day, the BMNC were washed twice with PBS buffer solution, followed by addition of 100 *μ*L bovine pancreatic ribonuclease (1 mg/mL) and incubation for 30 min in a 37°C water bath. The cells were finally stained with propidium iodide (PI, 50 *μ*L/mL) in the dark for 30 min at 4°C. The cells were analyzed using flow cytometry (Becton Dickinson, USA). The percentage of all cells was calculated using Cell Quest software (Becton Dickinson, USA).

### 2.6. Measurement of Apoptosis

After 1 to 14 days of treatment, BMNC suspension was isolated and stained with Annexin V-FITC/PI. The cellular apoptosis was analyzed with a flow cytometer (Becton Dickinson, USA). The steps outlined below were followed. Cells were washed twice with PBS and their concentration was adjusted to 1 × 10^6^/mL with 1x binding buffer; 5 *μ*L Annexin V-FITC and 5 *μ*L PI were added into 100 *μ*L samples. The samples were incubated for 15 min in the dark at 25°C after mixing followed by addition of 100 *μ*L 1x binding buffer to each sample. Apoptotic cells were distinguished with dual parameter analysis by flow cytometry and the apoptotic cell rate was calculated.

### 2.7. Detection of Expression of Adhesion Molecules CD44 and CD49d

After 1 to 14 days of treatment, 5 *μ*L of PE-anti-mouse Sca-1^+^ antibodies was added to 500 *μ*L of BMNC suspension, followed by incubation in the dark for 30 min at 4°C. Then 5 *μ*L FITC-anti-mouse CD44 antibodies or FITC-anti-mouse CD49d antibodies were added after washing with PBS and incubated in the dark for 30 min at 4°C. Cells were resuspended after a second wash and tested for the expression of CD44 or CD49d.

### 2.8. Analysis of SOD Activity and MDA Content

After 1, 7, and 14 days of treatment, BMNC suspension was added to 600 *μ*L of IP cell lysis solution placed in ice for 30 min and centrifuged at 12000 rpm for 5 min at 4°C. The supernatant was used for testing the protein content, SOD activity, and MDA content, according to the kit specifications.

### 2.9. Statistical Analysis

All data were expressed as the mean ± standard deviation (SD). Statistical significance was evaluated using Student's *t*-test and two-way ANOVA in the treatments. Differences were considered to be statistically significant if *P* < 0.05. All calculations and statistical analyses were performed using SPSS software for Windows version 19.0 (Chicago, IL, USA).

## 3. Results

### 3.1. Effect of LBP on Cell Cycle in BMNC of Mice Exposed to Ionizing Radiation Injury (Table 1, Figure 1)

**Figure 1:**
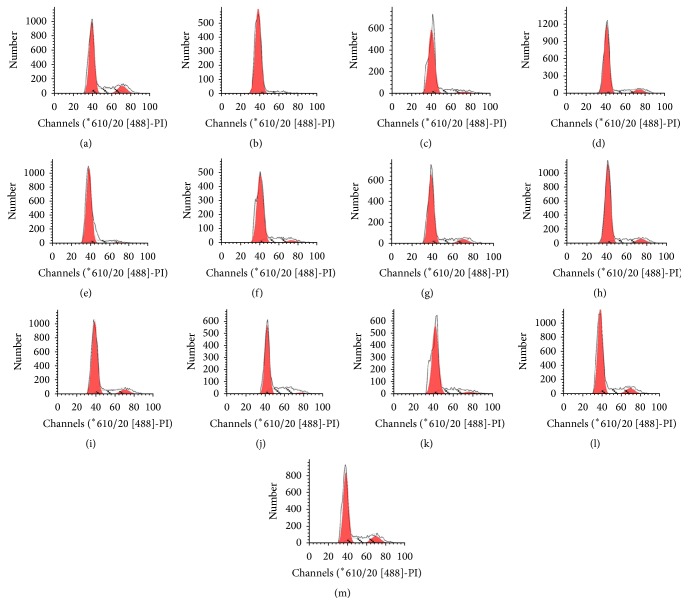
Typical flow cytometry histogram of BMNC cell cycle in mice: (a) normal control group, (b) NS group on day 1, (c) NS group on day 7, (d) NS group on day 14, (e) 50 mg/kg LBP group on day 1, (f) 50 mg/kg LBP group on day 7, (g) 50 mg/kg LBP group on day 14, (h) 100 mg/kg LBP group on day 1, (i) 100 mg/kg LBP group on day 7, (j) 100 mg/kg LBP group on day 14, (k) 200 mg/kg LBP group on day 1, (l) 200 mg/kg LBP group on day 7, and (m) 200 mg/kg LBP group on day 14. The first peak represents G_0_/G_1_ ratio.

Based on the DNA content, the cells were distributed in the corresponding peak. As shown in Figure 1, the first peak denotes G_0_/G_1_ ratio. The results from Table 1 indicate that the percentage of G_0_/G_1_ in NS group was higher than in the normal group (*P* < 0.05). Compared with the NS group, there were no significantly decreased G_0_/G_1_ ratios in 50 mg/kg LBP group.

The other LBP-treated irradiated mice showed a significant reduction in G_0_/G_1_ ratios compared with NS group, especially 14 d after treatment (*P* < 0.05). The G_0_/G_1_ ratios in the 200 mg/kg LBP group were the lowest.

### 3.2. Effect of LBP on Apoptosis in BMNC of Mice Exposed to Ionizing Radiation Injury (Table 1, Figure 2)

**Figure 2:**
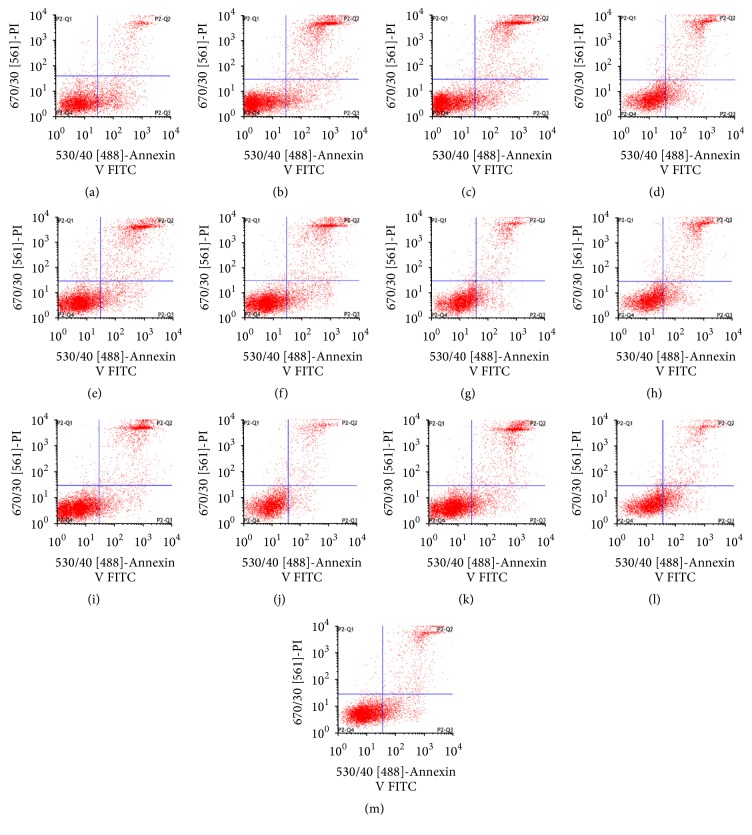
Typical flow cytometry histogram of apoptotic rate in BMNC: (a) normal control group, (b) NS group on day 1, (c) NS group on day 7, (d) NS group on day 14, (e) 50 mg/kg LBP group on day 1, (f) 50 mg/kg LBP group on day 7, (g) 50 mg/kg LBP group on day 14, (h) 100 mg/kg LBP group on day 1, (i) 100 mg/kg LBP group on day 7, (j) 100 mg/kg LBP group on day 14, (k) 200 mg/kg LBP group on day 1, (l) 200 mg/kg LBP group on day 7, and (m) 200 mg/kg LBP group on day 14.

Typical flow cytometry histogram of mouse apoptosis rate in BMNC is shown in Figure 2. These results from Table 1 showed that the percentage of apoptosis cells increased significantly in NS group, compared with the normal group (*P* < 0.05). The apoptosis rate in each LBP group decreased continuously over time. After 1 day, 7 days, and 14 days of treatment, the differences in LBP groups were statistically significant except 50 mg/kg LBP group (*P* < 0.05).

### 3.3. Effect of LBP on Sca-1^+^CD44 and CD49d Expression in BMNC of Mice Exposed to Ionizing Radiation Injury (Table 2)

As shown in Table 2, after 1 to 14 days of treatment, compared with normal controls, the expression of CD44 in the NS group was drastically increased on days 1, 7, and 14 after exposure (*P* < 0.05). Additionally, compared with NS group, on day 1, there were no significant decreases in each LBP group, but the differences on days 7 and 14 in 50 mg/kg LBP group, 100 mg/kg LBP groups, and 200 mg/kg LBP group were significant (*P* < 0.05). Compared with normal controls, the expression of CD49d in the NS group was also significantly increased on days 1, 7, and 14 after exposure (*P* < 0.05); the differences on each day in each LBP group were significant while compared with NS group (*P* < 0.05). However, the expression of CD44 and CD49d in LBP groups never attained the normal levels.

### 3.4. Effect of LBP on SOD Activity and MDA Content in BMNC of Mice Exposed to Ionizing Radiation (Figures 3 and 4)

**Figure 3:**
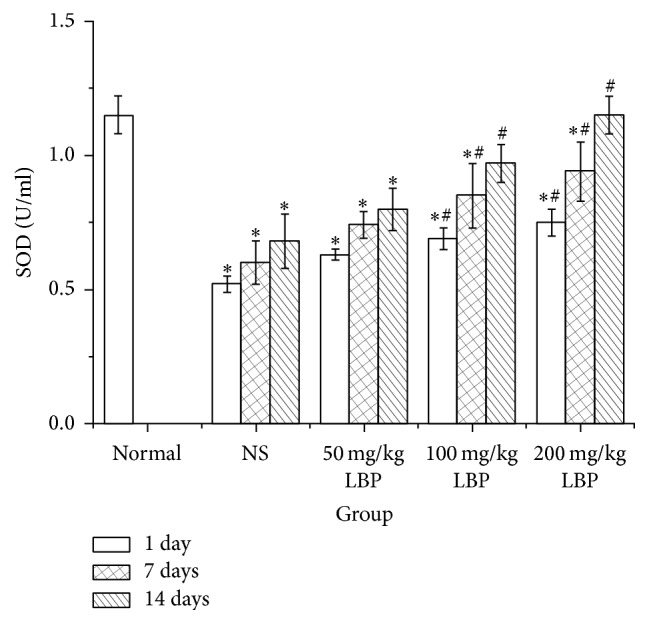
Effect of LBP on SOD in BMNC of mice exposed to ionizing radiation ^*∗*^ *P* < 0.05 versus normal control; ^#^ *P* < 0.05 versus NS group at the same speeding time.

**Figure 4:**
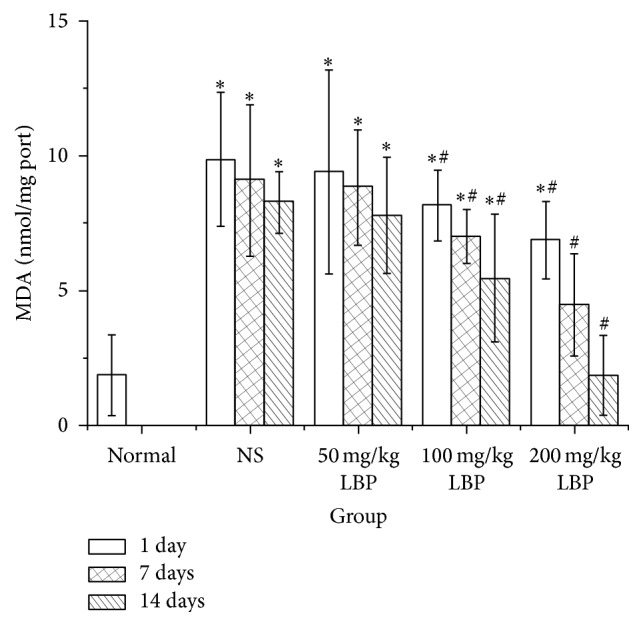
Effect of LBP on MDA in BMNC of mice exposed to ionizing radiation injury ^*∗*^ *P* < 0.05 versus normal control; ^#^ *P* < 0.05 versus NS group at the same speeding time.

As shown in Figure 3, after 1, 7, and 14 days of treatment, compared with normal control, treatment with NS induced a distinct decrease in SOD activity levels (*P* < 0.05). Compared with NS group, there was no significantly increased SOD activity in 50 mg/kg LBP group, but the differences in 100 mg/kg LBP groups and 200 mg/kg LBP group were statistically significant (*P* < 0.05). As seen in Figure 4, compared with the normal control, treatment with NS induced a distinct increase in the MDA levels (*P* < 0.05). Compared with the NS group, there was no significantly increased MDA level in 50 mg/kg LBP group. However, the differences in the 100 mg/kg LBP groups and the 200 mg/kg LBP group showed statistical significance (*P* < 0.05). During the observation, the SOD activity and the MDA content in NS group marginally recovered. However, the SOD activity and the MDA content in LBP groups failed to return to normal.

## 4. Discussion

Currently, electromagnetic pollution is recognized as one of the fourth largest pollutants after air, water, and noise pollution. LBP is a valuable Chinese medicinal herb, with a range of bioactivities. Studies indicate that LBP has antiradiation effects, most of which are related to the reproductive [2, 3, 23] and immune systems [5, 24]. Studies involving the role of radiation in hematopoietic system are few [26]. Therefore, we investigated the half-lethal dose of X-rays to establish animal models and study the influence of LBP on damage to the hematopoietic system after radiation.

Apoptosis is regulated genetically. We detected apoptotic rates and cell cycle using flow cytometry. The apoptotic rate is tested using Annexin V with PI staining. According to the DNA content, the cells at different cell cycle are determined. The first peak represents G_0_/G_1_ ratio. A large number of stimuli, both physiological and pathological, have been shown to induce apoptosis. Studies show that radiation induces cellular apoptosis [30–32]. In our study, flow cytometry data showed that the apoptotic rate and G_0_/G_1_ ratio in the NS group increased more significantly on days 1, 7, and 14 after irradiation than in the normal group, especially 1 d after irradiation. The result is consistent with other reports. After 1 to 14 days of treatment, the apoptotic rate and G_0_/G_1_ ratio in the LBP groups were markedly reduced. However, there were no statistically significant differences in the 50 mg/kg LBP group, perhaps because of inadequate dosage or duration of observation.

Treatment with a longer duration and larger LBP dose decreased the apoptotic rate drastically and significantly in the 100 mg/kg and 200 mg/kg LBP groups (*P* < 0.05). The radiation-induced damage was mediated by reduced apoptosis and G_0_/G_1_ ratio. The effect was more pronounced with increase in dosage and duration of exposure.

Adhesion molecules on cell surface mediate cell-cell and cell-matrix interactions. Most of them are glycoproteins involved in intracellular signal transduction and the regulation of cell migration, anchoring, proliferation, and differentiation. They also participate in various physiological and pathological processes, for example, embryonic development and differentiation, immune response, maintenance of normal structure, wound repair, blood clotting and invasion, and tumor metastasis. A large number of studies have shown that adhesion molecules such as CD44, CD49d, and CD34 facilitate the redistribution of hematopoietic stem cells and homing. CD44 is expressed in hematopoietic stem cells and other cells in the hematopoietic microenvironment. CD44 is a membrane receptor of hyaluronic acid, which plays a role in the structure and function of hematopoietic system [33]. The overexpression of CD44 is important in leukemia [34–37]. CD49d is another adhesion molecule. The combination of CD49d and chemokine receptors plays a significant role in the migration and homing of B cells in the bone marrow and lymphatic tissue [38]. Several studies show that the overexpression of CD49d is related to hematological disease [37, 39–41]. Blockage of CD49d expression enhances the treatment efficacy of hematopoietic disease [42]. In our experiment, compared with the mice in normal group, the expression of CD44 and CD49d in the NS group was increased significantly following exposure to ionizing radiation. During the 14-day observation, the expressions of CD44 and CD49d slightly decreased on day 14 in the NS group, maybe due to autoimmunization. After 1 to 14 days of treatment, the CD44 and CD49d expression in LBP group was markedly reduced. But, on day 1, the CD44 expression in each LBP group was not significantly decreased, maybe because the time of treatment is not long enough; the differences of CD49d in each LBP group were significant (*P* < 0.05). Increasing the duration and dosage of LBP suppressed the CD44 and CD49d expression significantly on days 7 and 14 (*P* < 0.05). Analysis of experimental results indicates that LBP provides resistance to radiation, in direct proportion to the dose and duration of treatment. However, normal levels are not restored, probably due to inadequate duration of observation or dosage.

Ionizing radiation induces free radical production, lipid peroxidation, and destruction of biological macromolecules such as nucleic acids, proteins, and enzymes, resulting in the destruction of cells and tissues [43–45]. SOD is an important antioxidant, which catalyzes disproportionate generation of superoxide anion radicals, effectively scavenging free radicals, to prevent any damage. It plays an important role in maintaining the redox balance. Free radicals and lipids generate MDA by peroxidation. MDA is cytotoxic and induces protein and nucleic acid crosslinking polymerization reactions. Therefore, MDA is a commonly used indicator in the physiology of senescence and resistance of animals and plants. MDA content is an indirect indicator of membrane damage. Studies have shown that LBP increases the activity of SOD, reduces the content of MDA, enhances the antioxidant capacity, and protects cells, resulting in antioxidative effects [2, 5]. In our experiment, compared with the mice in the normal group, the activity of SOD in NS group was notably decreased and the MDA content in the NS group was significantly increased. After 1, 7, and 14 days of treatment, the SOD activity in each group increased, and the MDA content in each group decreased. However, no statistically significant differences were seen in the 50 mg/kg LBP group, perhaps due to inadequate dosage or duration of observation.

The differences in 100 mg/kg LBP and 200 mg/kg LBP group were statistically significant. With the increase in dosage and duration of treatment, the SOD activity increased more obviously, and the MDA content decreased more prominently. Our study showed that LBP enabled the maintenance of the redox balance and protected cells against radiation damage.

The former results showed that LBP can reduce the DNA damage in hematopoietic system and promote the recovery of RBC and PLT and mild effects on WBC [26, 27]. But there have been few reports about the effects of on apoptosis, oxidative damage, and expression of adhesion molecules in BMNC of mice subjected to ionizing radiation injury. The effects of LBP on apoptosis, oxidative damage, and expression of adhesion molecules in BMNC may be one of the more important pharmacological factors of promoting hematopoiesis. However, there are still many problems that need to be resolved concerning plant polysaccharides; additional studies are needed to further establish the underlying mechanisms.

## 5. Conclusions

In short, our results show that LBP has a protective effect on radiation-induced damage; these new insights might be useful for investigating new antiradiation drugs which have fewer side effects.

## Figures and Tables

**Table 1:** Effect of LBP on cells cycle and apoptotic rate in BMNC of mice exposed to radiation injury (*n* = 6, *X* ± *s*).

Group	Days (d)	G_0_/G_1_ (%)	Apoptosis rate (%)
Normal control	—	65.63 ± 5.46	9.25 ± 0.93

NS group	1	84.80 ± 1.31^*∗*^	22.98 ± 1.09^*∗*^
	7	82.85 ± 2.74^*∗*^	21.13 ± 0.54^*∗*^
	14	78.16 ± 1.81^*∗*^	19.65 ± 2.31^*∗*^

LBP group			
50 mg/kg	1	82.33 ± 2.71^*∗*^	21.56 ± 1.07^*∗*^
	7	76.96 ± 4.10^*∗*^	19.24 ± 1.32^*∗*^
	14	74.57 ± 1.42	17.63 ± 1.15^*∗*^
100 mg/kg	1	77.18 ± 3.01^*∗*#^	20.67 ± 0.36^*∗*#^
	7	74.93 ± 3.77^#^	17.64 ± 1.92^*∗*#^
	14	70.84 ± 3.41^#^	12.37 ± 1.16^*∗*#^
200 mg/kg	1	76.32 ± 6.18^*∗*#^	19.65 ± 2.85^*∗*#^
	7	72.79 ± 3.75^#^	13.49 ± 2.64^*∗*#^
	14	67.85 ± 5.12^#^	10.64 ± 0.84^#^

^*∗*^ *P* < 0.05 versus normal control; ^#^ *P* < 0.05 versus NS group at the same feeding time.

**Table 2:** Effect of LBP on Sca-1+CD44 and CD49d expression in BMNC of injured mice exposed to ionizing radiation (*n* = 6, *X* ± *s*).

Group	Days (d)	CD44 (%)	CD49d (%)
Normal control	—	5.33 ± 0.77	3.42 ± 0.55

NS group	1	11.99 ± 1.35^*∗*^	11.85 ± 0.65^*∗*^
	7	11.78 ± 1.20^*∗*^	10.33 ± 1.39^*∗*^
	14	10.67 ± 1.09^*∗*^	8.70 ± 0.64^*∗*^

LBP group			
50 mg/kg	1	10.15 ± 0.66^*∗*^	9.23 ± 1.15^*∗*#^
	7	9.69 ± 0.90^*∗*#^	8.17 ± 1.57^*∗*#^
	14	8.41 ± 0.81^*∗*#^	5.30 ± 0.95^*∗*#^
100 mg/kg	1	9.78 ± 1.55^*∗*^	8.63 ± 0.74^*∗*#^
	7	8.96 ± 0.73^*∗*#^	6.33 ± 0.85^*∗*#^
	14	6.58 ± 0.74^#^	4.67 ± 0.72^#^
200 mg/kg	1	8.98 ± 1.37^*∗*^	7.99 ± 1.39^*∗*#^
	7	8.63 ± 0.73^*∗*#^	6.83 ± 1.10^*∗*#^
	14	5.50 ± 0.25^#^	3.78 ± 0.45^#^

^*∗*^ *P* < 0.05 versus normal control; ^#^ *P* < 0.05 versus NS group at the same speeding time.
